# Supplementary material for: Developing Device of Death Operation (DODO) to Detect Apoptosis in 2D and 3D Cultures
Source: Cells. 2024 Jul 20;13(14):1224. doi: 10.3390/cells13141224 (PMC11274962; doi:10.3390/cells13141224)
Supplement: Supplementary file 1 [file cells-13-01224-s001.zip › cells-3029875-supplementary.pdf]

Supplementary Information

Supplementary sequences

The amino acid sequence of mCherry-TEV_cleavage_site-RRRG:

MVSKGEEDNMAIIKEFMRFKVHMEGSVNGHEFEIEGEGEGRPYEGTQTAKLKVTKGG-PLPFAWDILSPQFMYGSKAYVKHPADIPDYLKLSFPEGFKWERVMNFEDGGVVTVTQDSSLQDGEFIYKVKLRGTNFPSDGPVMQKKTMGWEASSERMYPEDGALKGEIKQRLKLKDGGHYDAEVKTTYKAKKPVQLPGAYNVNI-KLDITSHNEDYTIVEQYERAEGRHSTGGMDELYKENLYFQSRRRG*

The amino acid sequence of mCherry-caspase3_cleavage_site-RRRG:

MVSKGEEDNMAIIKEFMRFKVHMEGSVNGHEFEIEGEGEGRPYEGTQTAKLKVTKGG-PLPFAWDILSPQFMYGSKAYVKHPADIPDYLKLSFPEGFKWERVMNFEDGGVVTVTQDSSLQDGEFIYKVKLRGTNFPSDGPVMQKKTMGWEASSERMYPEDGALKGEIKQRLKLKDGGHYDAEVKTTYKAKKPVQLPGAYNVNI-KLDITSHNEDYTIVEQYERAEGRHSTGGMDELYKGDEVDGRRRG*
